# Reviewing of Research Finding of Hepatitis B Virus Infection in Lao People’s Democratic Republic

**DOI:** 10.5005/jp-journals-10018-1265

**Published:** 2018-05-01

**Authors:** Angkham Ounavong

**Affiliations:** Department of Basic Health Sciences, Faculty of Medical Sciences, University of Health Sciences, Vientiane, Lao People’s Democrati Republic

**Keywords:** Occult infection donors, Serological study in different cohort, Seroprevalence of HBsAg among donors, Seroprevalence of HBsAg among pregnant women in Lao People’s Democratic Republic.

## Abstract

The seroprevalence of hepatitis B virus (HBV) surface antigen (HBsAg)-positive blood donors was 8.7%. The prevalence among males (9.7%) was higher than among females (6.2%). The prevalence of antihepatitis C virus (HCV)-positive blood donors was 1.1%, with no significant differences between males (1.1%) and females (1.0%). Annual positive rates for HBsAg and anti-HCV donors during the years 2003 to 2005 did not differ significantly. In Lao People’s Democratic Republic (PDR), HBV is highly endemic. However, blood donations are only screened for HBsAg, leaving a risk of transmission by HBsAg-negative occult infected donors. Here, we characterized first-time blood donors to assess prevalence of HBV infections and occult infected donors. Despite hepatitis B vaccination at birth and at 6, 10, and 14 weeks of age, HBV infection continues to be endemic in Lao PDR. We carried a cross-sectional serological study in infants, preschool children, school pupils, and pregnant women to determine their burden of disease, risk of infection, and vaccination status. The prevalence of HBsAg carriage in pregnant women is a relevant marker for the risk of mother-to-child HBV transmission. This study aimed to assess the changes in prevalence of HBV infection among pregnant women attending Mahosot Prenatal Clinic (Vientiane).

**How to cite this article:** Ounavong A. Reviewing of Research Finding of Hepatitis B Virus Infection in Lao People’s Democratic Republic. Euroasian J Hepato-Gastroenterol 2018;8(1):75-76.

## INTRODUCTION

There have been no previous reports of the prevalence of HBV and HCV infections in the general population of Lao PDR. Epidemiological data are necessary to determine the burden of disease that exists in the country. In order to estimate the seroprevalence of HBV and HCV infections in Lao PDR, a retrospective study of blood donors screened at the National Blood Transfusion Center, Lao Red Cross, Vientiane, from 2003 to 2005, was carried out. This report presents the results of screening for HBsAg and anti-HCV in this group of donors.^1^ In Western countries with a low prevalence of HBV infections, hepatitis B core antigen (anti-HBc) antibody testing is included in the screening of blood donations for transfusion-transmissible infection (TTI) to exclude individuals with past exposure to HBV [including the vast majority of occult HBV infection (OBI)].^2^ However, in highly endemic countries, this approach would exclude a large proportion of healthy donors that have cleared HBV infections, severely undermining the blood supply. In these settings, screening for TTI commonly includes HBsAg testing. However, this strategy does not detect OBI or acute infections during the initial infection period. The HBV is endemic in Lao PDR, about 8% of blood donors being chronic carriers of HBV, and is the cause of high morbidity and mortality.^3^ It is believed that most infections occur during early childhood, e.g., during birth or early family life. If infected at birth, children have a 90% risk of developing chronic infection. This rate decreases to 30% if infected between the ages of 1 and 5, and to 5 to 10% if infected after the age of 5. As screening for HBsAg in pregnant women^4^ and immunoglobulin prophylaxis in newborns are not widely practiced in Lao PDR, routine infant vaccination is the mainstay of the prevention of early childhood infection.

The first strategy was to vaccinate all children born to mothers infected with HBV. It involves a systematic screening for HBV markers of infection (HBsAg) and replication (HBeAg) in pregnant women, followed, if positive, by active-passive immunization of the newborn within the first 24 hours of life, combining HBV vaccine and hepatitis B immunoglobulin injected in two different sites.^5^ This strategy may reduce by 75 to 90% the mother-to-child HBV transmission, but it is difficult to generalize in highly endemic areas because of its high cost. For low-income countries, universal vaccination strategy for children without prenatal screening is the only way to control HBV infection and prevent its long-term sequelae.

## RESULTS

About 9.6% of the donors were HBsAg positive, and 45.5% were positive for at least one of the HBV serum markers. More than 40% HBsAg carriers were HBeAg positive, with HBeAg seroconversion occurring around 30 years of age. Furthermore, 10.9% of HBsAg-negative, anti-HBc, and/or anti-HBs-positive donors were occult infected with HBV. Thus, at least 3.9% of blood donations would potentially be unsafe, but HBV deoxyribonucleic acid (DNA) copy numbers greatly varied between donors. A low prevalence of HBsAg (0.5%) was detected among infants from Vientiane and Luang Prabang, indicating some success of the vaccination policy. However, only 65.6% had protective anti-HBs antibodies, suggesting that vaccination coverage or responses remain suboptimal, even in these urban populations. Of a total of 13,238 tested women of mean age of 26 years, 720 women [5.44% (95 confidence interval: 5.1-5.8%)] were found to be HBsAg positive, the annual prevalence ranging from 4.6 to 6.2%. A slight but steady and significant decrease in prevalence over the 7 years of the study could be documented.

## CONCLUSION

In Lao PDR, a sizable proportion of HBsAg negative and anti-HBc antibody-positive blood donations are potentially DNA positive and infective for HBV. Overall, the results demonstrate a dramatic deficiency in vaccination coverage and vaccine responses and/or documentation within the regions of Lao PDR studied, which included urbanized areas with better health care access. Timely and effective HBV vaccination coverage is needed in Lao PDR. Although below the 8% hyperendemic threshold, the HBsAg prevalence observed in pregnant women in Vientiane reflects a high risk of HBV perinatal transmission and calls for a widespread infant immunization with an HBV vaccine birth dose.
